# Effectiveness of Prophylactic Interventions in Neurogenic Heterotopic Ossification (NHO): A Systematic Review

**DOI:** 10.7759/cureus.27683

**Published:** 2022-08-04

**Authors:** Syed Muhammad Hannan Ali Rizvi, Joudi Sharaf, Kerry-Ann D Williams, Maha Tariq, Maitri V Acharekar, Sara Elena Guerrero Saldivia, Sumedha Unnikrishnan, Yeny Y Chavarria, Adebisi O Akindele, Ana P Jalkh, Aziza K Eastmond, Chaitra Shetty, Lubna Mohammed

**Affiliations:** 1 Research, California Institute of Behavioral Neurosciences & Psychology, Fairfield, USA

**Keywords:** non-steroidal anti-inflammatory drugs, electromagnetic field radiation, prophylaxis, bisphosphonate use, warfarin, traumatic brain injury, spinal cord injury, neurogenic heterotopic ossification

## Abstract

Neurogenic heterotopic ossification (NHO) is the formation of mature lamellar bone in peri-articular tissues following a neurological insult, most commonly traumatic brain injury (TBI) or spinal cord injury (SCI). NHO is a debilitating condition associated with significant morbidity and reduced quality of life. However, its pathophysiology remains poorly understood. While surgery is the mainstay of treatment once NHO has been diagnosed, prophylactic options are limited and not well studied. This review aimed to determine the efficacy of various interventions used in the primary prevention of NHO. We conducted an electronic literature search using five databases (PubMed, Embase, ScienceDirect, Cochrane Library, and Cumulative Index to Nursing and Allied Health Literature (CINAHL)) for records published until April 10, 2022. We identified 2,610 potentially eligible records across all databases. Nine reports met our eligibility criteria and were included in this review. Four were clinical trials (three randomized control trials, one nonrandomized trial), four were observational studies, and one was a systematic review/meta-analysis. The medications/interventions used included: warfarin, pulse low-intensity electromagnetic field therapy (PLIMF), bisphosphonates, and nonsteroidal anti-inflammatory drugs (NSAIDs). We did not find conclusive evidence to recommend the use of bisphosphonates and warfarin in the prevention of NHO. On the contrary, we found NSAIDs and PLIMF as effective prophylactic options based on the results of high-quality randomized control trials. Further prospective randomized studies with prolonged follow-ups are needed to confirm the long-term efficacy of these preventive interventions.

## Introduction and background

Heterotopic ossification (HO) is the abnormal presence of mature lamellar bone in an extra-skeletal site. Based on etiology, HO can be categorized as neurogenic, traumatic, or genetic (fibrodysplasia ossificans progressiva). Neurogenic heterotopic ossification (NHO) is HO secondary to neurological insult, most commonly after traumatic brain injury (TBI) or spinal cord injury (SCI) [1]. Although other neurological conditions such as Guillain-Barre syndrome [2,3], stroke [4,5], concussion [6], and Moyamoya disease [7] have been linked to NHO, the documented evidence is scarce.

The pathophysiology of NHO is poorly understood. It is generally agreed that a close nexus between nervous tissue injury and soft tissue inflammation is critical in the development of NHO. In recent years, the role of immune cells, particularly phagocytic macrophages, has been elucidated in the pathogenesis of NHO [8]. Understanding its pathogenesis is essential in identifying potential prophylactic strategies to reduce the need for complicated surgeries and morbidity.

NHO has an incidence of 10-23% in patients with TBIs and 10%-53% in patients with SCIs [9]. Clinical risk factors attributed to NHO include severity of CNS injury, spasticity, pressure ulcers, systemic infection, and prolonged immobilization [10]. The formation of HO occurs within three months of neurological injury, with a reported peak incidence in the second month [10]. It initially presents as pain, warmth, swelling, and decreased range of motion in the affected joint and, in many cases, leads to complete ankylosis of the joint [1]. Hips (60.9%) are the most common location, followed by elbows (21.3%), knees (14.3%), and shoulders (3.5%) [11]. Traditionally, NHO has been diagnosed using plain radiographs, bone scintigraphy, CT scan, and MRI. In the early phase of NHO, bone scintigraphy is positive with increased radionucleotide uptake and remains the gold standard for detecting early NHO [12]. In recent years, ultrasonography has been suggested as an alternative, safer, and cheaper option for early NHO detection [13].

Surgical excision is the mainstay of treatment for NHO once it develops. However, it creates a potential for added complications. While there is extensive literature on the treatment of NHO once it has been diagnosed, currently, there is no clinical consensus on prophylactic therapies. NHO is a debilitating complication that leads to significant morbidity and reduced quality of life. Therefore, it is imperative to identify effective options since prevention will potentially lead to faster rehabilitation and eliminate the need for surgeries. Herein, we present a systematic review to evaluate the effectiveness of various prophylactic interventions in NHO. 

## Review

Methods

We conducted this systematic literature review in accordance with the Preferred Reporting Items for Systematic Reviews and Meta-Analyses (PRISMA) 2020 guidelines [14].

Search Strategy

We thoroughly searched through PubMed (MEDLINE), Embase, Cochrane Library, ScienceDirect, and Cumulative Index to Nursing and Allied Health Literature (CINAHL) for studies published until April 10, 2022. We used a standard search strategy in PubMed, incorporating appropriate keywords and Medical Subject Heading (MeSH) terms via the Boolean method. We modified the strategy according to each database to obtain the most relevant results. In addition, we performed a manual search using reference screening and citation tracking of included studies to retrieve any reports that might have been missed from our initial search. Grey literature was not explored in this review. The literature search was conducted in April 2022, and all records were collected into one Endnote library. Table 1 highlights the keywords used in our search strategy.

**Table 1:** Search strategy CINAHL: Cumulative Index to Nursing and Allied Health Literature

Database	Search terms/Keywords
PubMed	Heterotopic ossification OR ("Ossification, Heterotopic"[Majr] OR "Ossification, Heterotopic/therapy"[Majr] OR "Ossification, Heterotopic/drug therapy"[Majr] OR "Ossification, Heterotopic/radiotherapy"[Majr] OR "Ossification, Heterotopic/surgery"[Majr] OR) AND (Traumatic brain injury OR "Brain Injuries, Traumatic"[Mesh] OR "Brain Injuries, Traumatic/complications"[Mesh] OR Spinal cord injury OR "Spinal Cord Injuries"[Mesh])
Embase	('spinal cord injury'/exp OR 'spinal cord injury' OR (spinal AND cord AND ('injury'/exp OR injury)) OR 'traumatic brain injury'/exp OR 'traumatic brain injury' OR (traumatic AND ('brain'/exp OR brain) AND ('injury'/exp OR injury))) AND ('heterotopic ossification'/exp OR 'heterotopic ossification') AND [english]/lim AND [humans]/lim AND ([embase]/lim OR [medline]/lim)
Cochrane Library	Heterotopic ossification OR HO
ScienceDirect	Heterotopic ossification AND (spinal cord injury OR traumatic brain injury)
CINAHL	Heterotopic ossification AND (spinal cord injury or SCI or spinal injury OR traumatic brain injury or head injury, or brain injury or TBI)

Inclusion and Exclusion Criteria

We formulated our eligibility criteria based on the population, intervention, comparison, and outcome (PICO) framework. We limited our search to records published in English, available as full-texts, and human subjects only. We included studies that met the following inclusion criteria: 1) studies directly comparing prophylactic modality to placebo for primary prevention of NHO following either SCI or TBI, 2) NHO was diagnosed by either bone scintigraphy, x-ray, or clinical signs, and 3) studies reporting the incidence of NHO for patients receiving intervention or placebo. We restricted our choice of study to systematic reviews/meta-analyses, clinical trials, and observational cohorts. We excluded studies based on the following criteria: 1) primary prophylaxis of NHO was not the main focus of the study, 2) studies focused on secondary prevention of NHO after surgical removal of heterotopic bone, 3) participants in the study were of nonneurogenic etiology, 4) any neurogenic etiology other than SCI or TBI, and 5) case reports/case series, editorials, conference abstracts, and studies published in languages other than English. 

Screening and Study Selection

We de-duplicated results manually and via Endnote. References remaining after this step were exported to Rayyan.ai for the screening process. Two of us (S.M.H.A.R. and J.S.) individually assessed and decided on each abstract's inclusion in our study based on the defined eligibility criteria. We decided if the articles were to be included (Yes), excluded (No), or needed further examination of full text (Maybe). The decisions were then compared; all double "Yes" articles were set aside in the inclusion category, all double "No" articles were excluded, all double "Maybe" articles were carried to the next full-text round. We sought the opinion of a third reviewer (K.W.) to resolve disagreements. From a total of 2,610 articles initially identified, nine reports were deemed eligible for inclusion in our review. The reports included one systematic review/meta-analysis, four clinical trials (three randomized control trials, one nonrandomized trial), and four observational studies.

Data Extraction

Two researchers (S.M.H.A.R. and J.S.) performed data extraction to ensure data quality and accuracy. We extracted the key characteristics of each eligible study and inserted them in tables in Microsoft Word to facilitate the analysis and presentation. We extracted the following variables from each study: first author and year of publication, country of origin, study overview (i.e., study design, methodology, study population, sample size, intervention used, drug regimen, time-to-treatment, length of treatment, duration of follow up, and assessment of NHO), and study findings reported using relative risk (RR) and odds ratio (OR) along with the 95% confidence intervals (CIs), and p-values.

Quality Appraisal

The quality assessment of each study was conducted separately by two of us (S.M.H.A.R. and J.S.) using appropriate quality appraisal tools. The opinion of a third reviewer was sought in case of any disagreements. For studies to be eligible, they had to score at least 70% or have an overall low risk (LR) of bias depending on the quality appraisal tool used. Using the Cochrane risk of bias 2 (RoB 2) tool, we assessed the potential RoB for randomized control trials in five domains. For each domain, the RoB was scored as either low, high, or unclear. Studies were characterized as having low, high, or unclear overall RoB based on the following criteria: low overall risk studies had a high risk (HR) of bias in two or less than two domains; high overall risk studies had a HR of bias in more than two domains; unclear overall risk studies had an unclear risk (UC) of bias in more than two domains. Table 2 shows the results of Cochrane RoB 2 tool.

**Table 2:** Assessment of clinical trials using the revised Cochrane RoB 2 tool RoB: risk of bias; LR: low risk; UC: unclear; HR: high risk

First author (year)	Random allocation	Intervention nonadherence	Incomplete results	Inadequate assessment of the outcomes	Selective reporting	Final RoB judgment
Banovac et al. (2001) [15]	LR	LR	LR	LR	LR	LR
Banovac et al. (2004) [16]	LR	LR	LR	LR	LR	LR
Durovic et al. (2009) [17]	UC	LR	LR	LR	LR	LR

Similarly, the quality appraisal of observational studies and nonrandomized clinical trials was performed using the Newcastle-Ottawa Scale (NOS). Each study was scored for eight items within three domains: subject selection, comparison, and results. The total points possible were four points for selection, two for comparability, and three for results. We interpreted the sum of scores as either good, fair, or poor quality based on the Agency for Healthcare Research and Quality (AHRQ) standards. Table 3 summarizes the quality assessment of included studies using the NOS tool.

**Table 3:** Newcastle-Ottawa risk-of-bias tool results for included observational studies 1 ★ represents one point NOS: Newcastle-Ottawa Scale; AHRQ: Agency for Healthcare Research and Quality

Author (Year)	Selection (/4)	Comparison (/2)	Results (/3)	Final NOS Score (/9)	AHRQ Standards
Stover et al. (1976) [18]	★★★	★	★★★	7	Good
Spielman et al. (1983) [19]	★★★★	★	★★★	8	Good
Buschbacher et al. (1992) [20]	★★★★	★	★★	7	Good
Ploumis et al. (2015) [21]	★★★★	★	★★★	8	Good
Zakrasek et al. (2019) [22]	★★★★	★	★★★	8	Good

Finally, we assessed the quality of systematic reviews/meta-analyses using the Assessment of Multiple Systematic Reviews 2 (AMSTAR 2) tool. The AMSTAR 2 tool consisted of 16 items that were scored as either Yes, Partial Yes, or No. We rated the study quality as either critically low, low, moderate, or high based on a score out of 16. Table 4 summarizes the AMSTAR 2 tool results.

**Table 4:** AMSTAR 2 checklist results for included systematic reviews AMSTAR 2: Assessment of Multiple Systematic Reviews 2; Y: Yes; PY: Partially Yes; N: No

First Author (Year)	Item 1	Item 2	Item 3	Item 4	Item 5	Item 6	Item 7	Item 8	Item 9	Item 10	Item 11	Item 12	Item 13	Item 14	Item 15	Item 16	Overall Quality
Yolcu et al. (2020) [23]	Y	N	Y	PY	Y	Y	Y	Y	Y	N	Y	Y	Y	Y	Y	N	Moderate

Results

**Figure 1:**
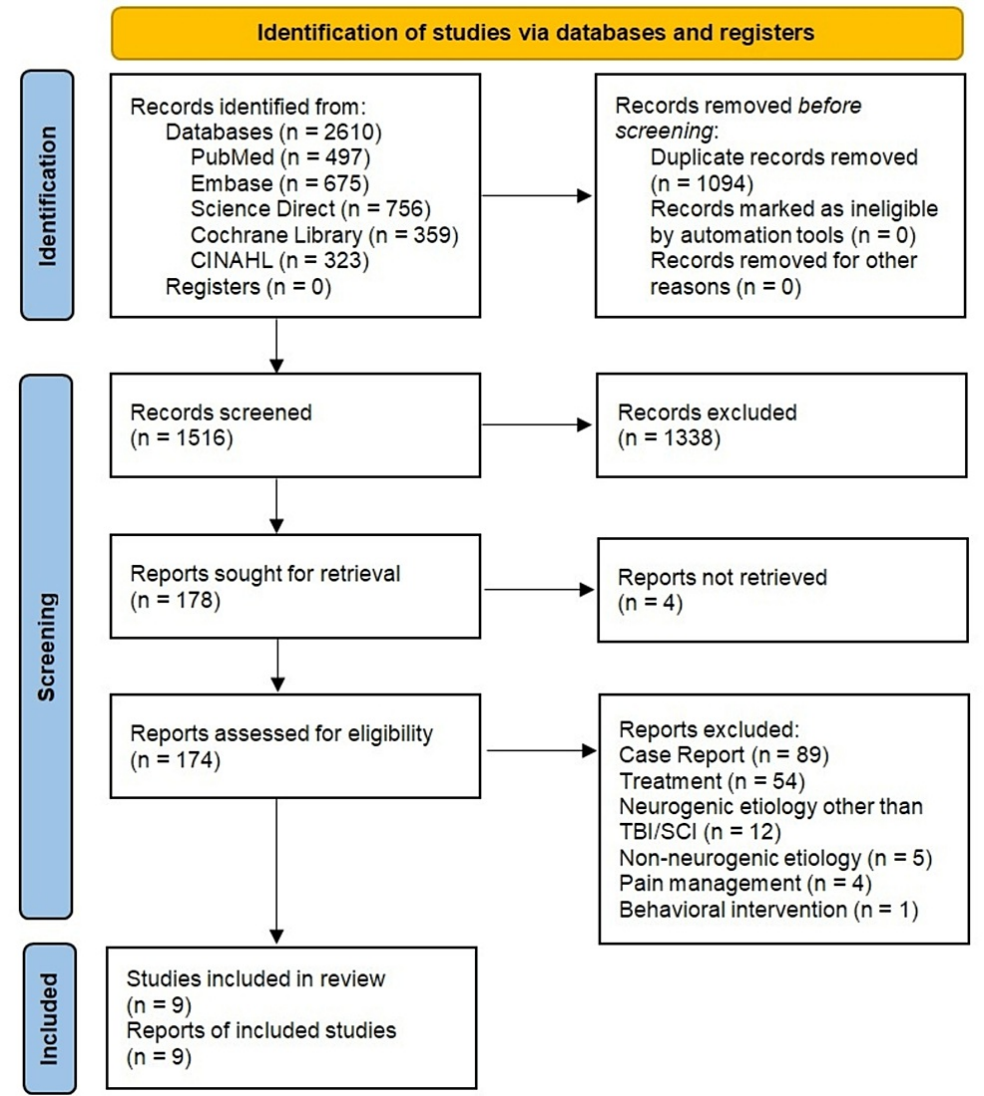
PRISMA flow diagram PRISMA: Preferred Reporting Items for Systematic Reviews and Meta-Analyses; CINAHL: Cumulative Index to Nursing and Allied Health Literature; TBI: traumatic brain injury; SCI: spinal cord injury

We identified a total of 2,610 studies through our initial search. Out of 2,610 records, 497 originated from PubMed, 675 from Embase, 756 from Science Direct, 359 from the Cochrane library, and 323 from CINAHL. After discarding duplicates, the remaining 1,516 articles were screened based on their title/abstract. A total of 178 reports were sought for full-text review. Of these, nine studies met our eligibility criteria and were included in this review. Four studies were clinical trials (three RCTs, one nonrandomized trial), four were observational studies, and one was a systematic review/meta-analysis. The medications/interventions used included: warfarin, pulse low-intensity electromagnetic field therapy (PLIMF), bisphosphonates, and nonsteroidal anti-inflammatory drugs (NSAIDs). The complete PRISMA flow diagram of our systematic review is provided in Figure 1. Table 5 and Table 6 outline the characteristics and details of the included studies, respectively.

**Table 5:** Characteristics of studies included in the review RCT: randomized control trial; SCI: spinal cord injury; TBI: traumatic brain injury; PLIMF: pulse low-intensity electromagnetic field therapy; EHDP: etidronate; ALN: alendronate; NSAIDs: nonsteroidal anti-inflammatory drugs; N/A: not available

Author (year)	Country	Study design	Population	Patient number		Medication/Intervention	Time to treatment (average)	Length of treatment	Assessment of NHO	Follow-up time
				Treatment	Placebo					
Buschbacher et al. (1992) [20]	USA	Observational	SCI	33	228	Warfarin	5.4 weeks post injury	N/A	Bone scan and/or x-rays	12.5 weeks
Durovic et al. (2009) [17]	Italy	RCT	SCI	14	15	PLIMF	7 weeks post injury	4 weeks	Plain radiography (x-rays)	12 weeks
Stover et al. (1976) [18]	USA	Nonrandomized trial	SCI	74	75	EHDP	58 days (8.2 weeks) 20-120 days post injury	8-12 weeks	Plain radiography (x-rays)	9 months
Spielman et al. (1983) [19]	USA	Observational	TBI	10	10	EHDP	2-7 days post injury	6 months	Plain radiopraphy (x-rays)	24 months
Ploumis et al. (2015) [21]	USA	Observational	SCI	125	174	ALN	21.8 days post injury	38.17 +/- 57.89 days	Bone scintigraphy + x-rays	1.71 years, minimum 3 months
Banovac et al. (2001) [15]	USA	RCT	SCI	16	17	Indomethacin	21 +/- 14 days	3 weeks	Bone scintigraphy + x-rays	6 months
Banovac et al (2004) [16]	USA	RCT	SCI	37	39	Rofecoxib	24 days	4 weeks	Clinical signs and symptoms, bone scintigraphy, and x-rays	N/A
Zakrasek et al. (2018) [22]	USA	Observational	SCI	27	81	Indomethacin/Celecoxib	Within 60 days post injury	>15 days	Clinical signs and symptoms, bone scintigraphy, CT scan, and x-rays	63 days
Yolcu et al. (2020) [23]	USA	Systematic review/Meta-analysis	SCI	257	558	NSAIDs, Bisphosphonates, Warfarin	21.8-58 days	Variable	X-rays, bone scintigraphy, or clinical signs	Variable

**Table 6:** Details of studies incorporated in the systematic review NHO: neurogenic heterotopic ossification; RCT: randomized control trial; SR/MA: systematic review/meta-analysis; SCI: spinal cord injury; DVT: deep venous thrombosis; PE: pulmonary embolism; ROM: range of motion; PLIMF: pulse low-intensity electromagnetic field therapy; EHDP: etidronate; ALN: alendronate; OD: once daily; TD: thrice daily; RR: relative risk; OR: odds ratio; CI: confidence interval

Author (year)	Methods	Drug regimen/Intervention frequency	Outcomes and measures	Results
Buschbacher et al. (1992) [20]	Observational (Retrospective): Information from discharge summaries, inpatient and outpatient data of 227 patients was gathered. 33 Patients were treated with warfarin; 94% of study participants were male, while 6% were female, with an average age of 34 years.	Patients had been treated with warfarin for approximately 5.4 weeks post-SCI injury for either DVT or PE. The dosage of warfarin was not mentioned.	Not mentioned. Bone scintigraphy and/or x-rays were done only if clinical signs raised suspicion of NHO.	None of the patients who were administered warfarin (n=33) developed NHO. 34 out of 194 (17.5%) patients who were not treated with warfarin, developed NHO. A statistically significant (p<0.01) inverse relationship was found between warfarin administration and formation of NHO.
Durovic et al. (2009) [17]	RCT: 29 patients were randomly divided into treatment and control groups. 14 patients were treated with PLIMF, exercise and ROM therapy; 15 patients were only treated with exercise and ROM therapy. Study included individuals aged 18-45 years. 28 participants were male, while only one was female.	Patients in the treatment group underwent PLIMF therapy five times a week for four weeks. Characteristics of PLIMF therapy: induction of 10 miliTesla (mT), frequency of 25 Hz, and duration of 30 min using the apparatus "Magnemed MT-91, Electromedicina Nis".	Incidence of NHO, measured using x-rays.	None of the patients from treatment group (n=14) had developed NHO by the end of therapy. Five out of 15 (33.3%) patients in the control group developed NHO. Incidence of NHO differed significantly between treatment and control groups (p<0.04).
Stover et al. (1976) [18]	Nonrandomized trial: 74 patients were treated with EHDP, while 75 patients were given a placebo. All participants in the study were male and above 16 years of age.	20 mg/kg/day EHDP for first two weeks, followed by 10 mg/kg/day EHDP for remaining treatment period.	Incidence of NHO using plain radiographs (x-rays).	Six patients out of 58 (10%) who were negative prestudy developed NHO in the treatment group; while 12 patients out of 56 (21%) who were negative prestudy developed NHO in the control group. A statistically significant (p<0.05) reduction in NHO incidence was observed in the treatment group compared to the control group.
Spielman et al. (1983) [19]	Observational cohort: 10 patients were treated with EHDP, while 10 patients comprised the nontreatment group. Mean age of participants was 31 years for the treatment group, and 27 years for the nontreated group. 16 participants were male, while four were female.	20 mg/kg/day EHDP for the first 12 weeks, followed by 10 mg/kg/day for the second 12 weeks.	Incidence of NHO using plain radiographs (x-rays).	Two out of 10 (20%) patients who received EDHP developed NHO, while seven out of 10 (70%) patients who received no treatment were found to have NHO. Incidence of NHO differed significantly between treatment and placebo group (p<0.025).
Ploumis et al. (2015) [21]	Retrospective database review: Clinical data of 299 patients was extracted. 125 patients received oral ALN while 174 patients did not receive oral ALN. 226 participants were male, female were 73. Mean age of patients was 42.7 +/- 18.36 years.	Oral 70 mg ALN was prescribed weekly for an average of 38.17 +/- 57.89 weeks.	Primary outcome measure was the incidence of NHO using mostly x-rays and rarely bone scintigraphy. Secondary outcomes measured included time of NHO appearance post-injury, affected joint, and type of treatment.	Seven patients out of 125 who received ALN were diagnosed with NHO. 12 patients out of 174 who did not receive ALN were found to have NHO. No significant correlation was found between the diagnosis of NHO and ALN intake. OR (95% CI) of not developing NHO versus NHO following treatment with ALN was 0.8 (0.3-2).
Banovac et al. (2001) [15]	RCT: 33 patients were randomly divided into treatment and control groups. 16 patients were treated with indomethacin for three weeks while 17 patients were given placebo. Study was discontinued and patients were initiated on EHDP in case of positive bone scan for NHO. Mean age of the patients was 33 years; all the participants were male.	Oral slow-release indomethacin 75 mg daily for three weeks.	Incidence of NHO using bone scintigraphy and x-rays.	Four out of 16 (25%) patients in the treatment group showed early NHO on bone scintigraphy compared to 11 out of 17 (65%) patients in the control group. Similarly, two out of 16 patients (13%) in the treatment group showed radiographic evidence of late NHO compared to seven out of 17 (41%) patients in the control group. There was a significantly lower incidence of both early NHO (p<0.001) and late NHO (p<0.001) in the indomethacin-treatment group compared to control.
Banovac et al (2004) [16]	RCT: 76 patients were randomly divided into treatment and control groups. 37 patients received rofecoxib while 39 patients were given placebo. Mean age of participants was 32 years, 11 were female, and 65 were male.	Oral rofecoxib 25 mg daily for four weeks.	Incidence of NHO using clinical signs and symptoms, bone scintigraphy, and x-rays.	Only five out of 37 (13.4%) patients in the treatment group developed NHO, compared to 13 out of 39 (33.3%) patients in the control group (p<0.05). Patients treated with Rofexoxib had 2.5 times lower RR of developing NHO than patients in the control group. (95% CI, 2.3-6).
Zakrasek et al. (2018) [22]	Retrospective: Clinical data of 108 patients was collected through chart review. 27 patients were treated with NSAIDs, while 81 did not receive any prophylaxis.	Patients were treated with either indomethacin 75 mg sustained-release OD, 25 mg immediate-release TD, or celecoxib 200 mg OD. Length of treatment varied among patients, but the study included only patients who had been given >15 days of prophylaxis.	Incidence of NHO	Two out of 27 (7.4%) patients treated with NSAIDs were diagnosed with NHO, while 29 out of 81 (35.8%) patients in the nontreatment group were diagnosed with NHO. Logistic regression analysis was applied. Patients who received >15 days of prophylaxis had an OR of 0.1 of being diagnosed with NHO compared to the control group (95% CI, 0.02-0.05).
Yolcu et al. (2020) [23]	SR/MA: A literature search was conducted using Embase, Ovid Medline, Scopus, EBM, and Web of Science. Five studies were included: 257 people comprised the prophylactic group, while 558 were in the nontreatment group. Incidence of NHO was pooled and compared between the treatment and placebo group. Meta-analysis was performed using Revman. Subgroup meta-analysis focusing on NSAIDs and bisphosphonates was also performed.	N/A	Incidence of NHO	In the overall analysis, no statistically significant difference was found between treatment group and placebo (RR (95% CI): 0.53 (0.26, 1.11); p=0.09). In the NSAID subgroup, those who received prophylaxis showed significantly lower incidence of NHO (RR (95% CI): 0.35 (0.19,0.16); p<0.001). In the bisphosphonate subgroup, no statistically significant difference was found (RR (95% CI): 0.65 (0.26, 1.64); p=0.58).

Discussion

**Figure 2:**
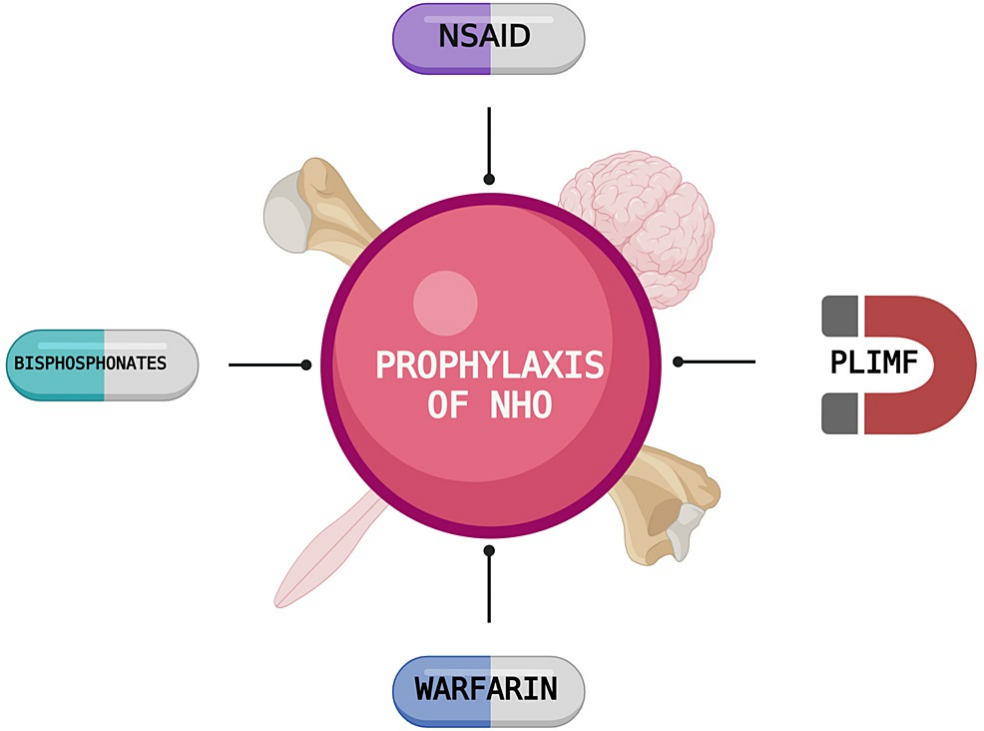
Prophylaxis of NHO Figure created with BioRender.com NHO: neurogenic heterotopic ossification; NSAID: nonsteroidal anti-inflammatory drug; PLIMF: pulse low-intensity electromagnetic field therapy

In this section, we will discuss the pathogenesis of NHO and the different interventions used for its prevention. The main pharmacological options include Warfarin, bisphosphonates, and NSAIDs.The only nonpharmacological therapy included in this study is PLIMF. Figure 2 illustrates the various options.

Pathogenesis

According to Chalmers et al. [24], three conditions need to be present for HO to occur: 1) The presence of osteoprogenitor cells (OPCs), 2) an osteoinductive factor, and 3) a permissive environment favoring osteogenesis. Despite continued efforts to identify the triggering molecular events leading up to NHO, the exact pathogenesis remains unclear. Here we present new evidence and hypotheses on the mechanisms that may lead to NHO formation.

NHO appears to form similarly following both TBI and SCI [25]. It has been hypothesized that a combined insult of CNS injury and local inflammation is a pre-requisite for ectopic bone formation at peripheral sites. The absence of either of these triggers is insufficient in causing NHO [26]. The animal models in recent years support this theory. Genêt et al.' s [27] animal model using genetically unmodified mice showed that cardiotoxin (CDTX)-mediated muscular injury alone did not cause the formation of HO. However, when combined with SCI, CDTX-mediated muscular injury resulted in clinically recognizable NHO within one-to-three weeks. Brady et al. [28], in their novel rat model, showed that a combination of TBI, femoral fracture, and muscle crush injury resulted in NHO development six weeks post-injury in 70% of experimental mice. Similarly, in their rat model, Wei‑Zhe et al. [29] demonstrated that TBI combined with Achilles tendon rupture resulted in pathological bone tissue formation in local tissue.

Damage to the CNS and peripheral tissue initiates an inflammatory cascade that leads to the release of pro-osteoinductive factors and the recruitment of inflammatory cells, particularly macrophages. Some of the factors implicated in the pathogenesis of NHO include substance P, oncostatin M (OSM), calcitonin gene-related protein (CGRP), bone morphogenetic protein-2 (BMP-2), fibroblast growth factors (FGFs), interleukin-1 (IL-1), and transforming growth factor beta (TGF-β1) [30,31]. The mechanism by which macrophages promote NHO involves activating the OSM signal transducer and activator of transcription (STAT-3) signaling pathway [26]. Accumulated OSM derived from activated macrophages promotes osteogenic differentiation of mesenchymal OPCs [32]. In addition, macrophages also secrete matrix metalloproteinase 9 (MMP-9), which mediates cell migration and extracellular matrix (ECM) degradation and remodeling, thus creating a conducive environment for OPCs differentiation [29]. This leads to the proliferation of fibroadipogenic progenitors (FAPs) within the injured periarticular muscles and their subsequent osteogenic differentiation. FAPs are derived from mesenchyme [9] and can differentiate into chondrocytes, osteoblasts, or adipocytes [33]. Hypoxia appears to be one of the main factors contributing to a conducive environment. It promotes chondrocyte differentiation by up-regulating chondrocyte-specific gene expression under the influence of hypoxia-inducing factor 1 alpha (HIF-1 α) [34]. Using animal models, Tannous et al. [35] established that HO follows the same path as endochondral ossification. In endochondral ossification, chondrocytes form a cartilaginous model of bone that is replaced by osteoblasts and osteoclasts with a woven bone matrix and eventually remodeled to lamellar bone.

**Figure 3:**
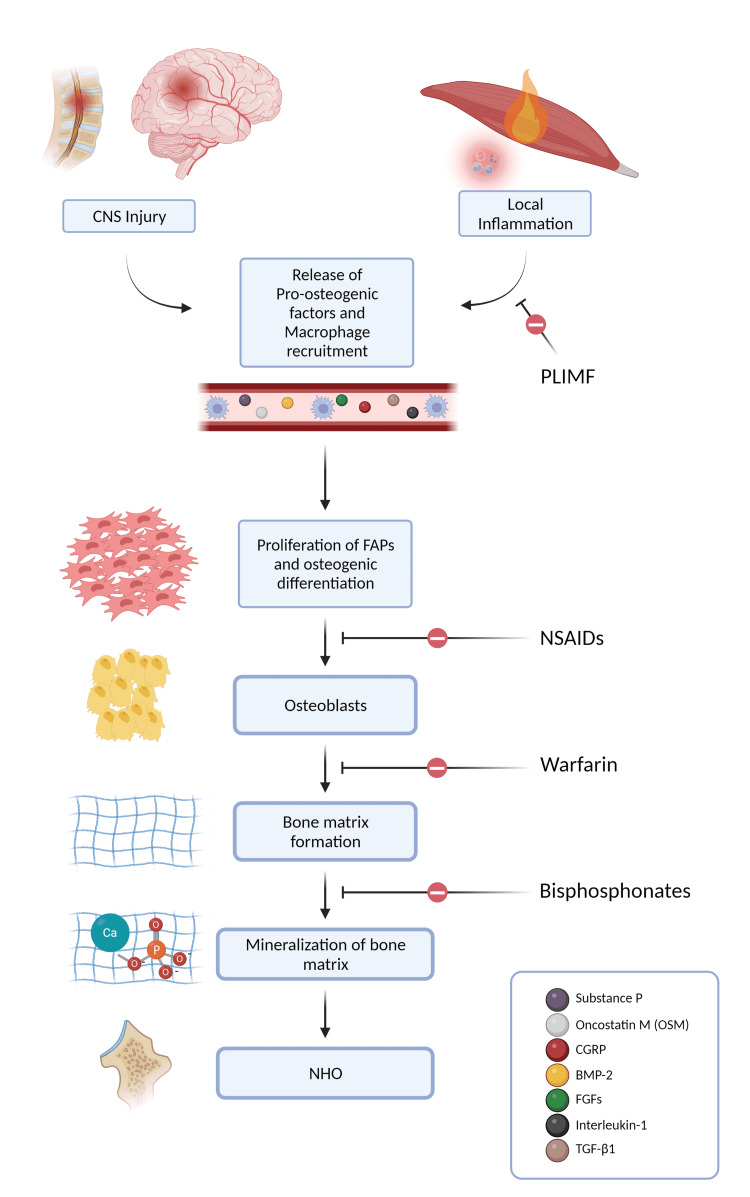
Pathogenesis of NHO Figure created with BioRender.com NHO: neurogenic heterotopic ossification; OSM: oncostatin M; FAPs: fibro-adipogenic progenitors; CGRP: calcitonin gene-related protein; BMP-2: bone morphogenetic protein-2; FGFs: fibroblast growth factors; IL-1: interleukin-1; TGF-β1: transforming growth factor-beta; PLIMF: pulse low-intensity electromagnetic field therapy; NSAIDs: nonsteroidal anti-inflammatory drugs

Recent research has sparked exciting ideas that could lead to the discovery of novel biomarkers and new therapeutic options for preventing NHO. Figure 3 summarizes the pathogenesis of NHO.

Warfarin

Warfarin has been proposed as potential prophylaxis for NHO based on the findings of a single observational study, which reported a significant association between the administration of warfarin post-SCI and failure to develop NHO [20]. In their study, none of the 33 patients treated with warfarin were diagnosed with NHO, while 34 out of 194 patients in the nontreatment group developed clinical signs of NHO. Warfarin is a commonly used anticoagulant that inhibits vitamin K epoxide reductase (VKOR). VKOR reduces vitamin K 2,3-epoxide and vitamin K to hydroquinone, an essential cofactor for the gamma-carboxylation of vitamin K-dependent clotting factors II, VII, IX, X, and proteins C, S [36]. Not only is y-carboxylation necessary for the function of several coagulation factors, but it is also known to regulate matrix Gla-protein (MGP), which inhibits extra-osseous tissue ossification, and osteocalcin/bone Gla-protein (BGP) [37]. Osteocalcin is a noncollagenous protein that comprises 1-2% of total protein in bone. Buschbacher et al. [20] suggested that warfarin, through its role in the depletion of reduced vitamin K, could be utilized to inhibit the carboxylation of osteocalcin, thereby preventing bone matrix formation and mineralization. While initially osteocalcin was thought to play a role in bone mineralization, research in recent years suggests it plays no part in bone mineralization. In fact, it plays a role in the regulation of glucose homeostasis [38]. The mechanism of action of warfarin in preventing NHO remains unknown and further research is needed to investigate this ostensibly protective effect against NHO before recommending its use. It should be noted that warfarin has a considerable side effect profile, mainly bleeding, and is not a suitable agent in many cases. 

PLIMF Therapy

The use of PLIMF therapy as prophylaxis for NHO is supported by a single RCT [17], which reported a significant difference in NHO incidence between treatment and control groups. None of the participants in the treatment group were diagnosed with NHO, whereas 33% of individuals in the control group developed NHO. PLIMF therapy uses a solenoid apparatus ("Magnamed MT-91, Electromedicina Nis") that employs electromagnetic fields. Durovic et al. [17] theorized that by increasing blood flow and oxygenation to areas of inflammation and removing toxic substances, PLIMF therapy could potentially eliminate the neurogenic stimulus required for NHO pathway activation. Although this study demonstrated the efficacy and safety of PLIMF therapy in preventing NHO, there are concerns over its feasibility. Further research is warranted with larger sample size and longer follow-up duration to evaluate its long-term efficacy.

Bisphosphonates

Bisphosphonates are used in a multitude of skeletal disorders, including osteoporosis, Paget disease of bone, and metastatic bone disease. They are pyrophosphate analogs that inhibit osteoclastic bone resorption by binding hydroxyapatite in bone [39]. Two bisphosphonates, alendronate (ALN) and disodium etidronate (Didronel/EHDP), have been studied as therapeutic agents in the prevention of NHO. Stover et al. [18], theorized that EHDP may have an inhibitory effect on the pathogenesis of HO by inhibiting bone mineralization. In their prospective, double-blind study of individuals with SCI, earlier treatment with EHDP resulted in a significantly lower incidence of NHO (p<0.05). Based on their findings, there is some evidence to suggest that EHDP treatment begun within 60 days post SCI is more beneficial than when it is started more than 60 days after injury. Minor gastrointestinal symptoms were noted in their study, such as nausea, vomiting, and diarrhea.

Similarly, Spielman et al. [19] sought to determine the prophylactic effect of EHDP in decreasing the incidence of HO in the TBI population. The difference in NHO incidence between the study and control groups was found to be significant in their study (p<0.025). Interestingly, in their study, the most common locations affected were elbows (35%), shoulders (29%), hips (18%), and knees (18%). This is in contrast to patients in the spinal cord population in which NHO seems to develop mainly in the hip region. Despite the preliminary findings strongly suggesting that initiating EHDP therapy during the acute phase of both spinal cord and severe head injury decreases NHO incidence, these findings should be interpreted with caution due to the small sample size and the noncontrolled nature of the study. Even though fewer patients treated with EHDP developed NHO during the eight-to-12 weeks of study, the overall prevalence may not have differed much a year later. Furthermore, it should be noted that EHDP is no longer available in the USA.

ALN is a potent N-containing second-generation bisphosphonate that is thought to inhibit osteoclastic activity and have a minor effect on bone mineralization [21]. In comparison to EHDP, ALN has a higher antiosteoporotic effect. Ploumis et al. [21] reported no statistically significant reduction in the incidence of NHO in patients treated with ALN. While there was no direct correlation between the prevention of NHO and ALN intake, there seemed to be an indirect connection between the two, as serum alkaline phosphatase (ALP) levels were abnormally raised in patients with NHO whilst patients who took oral ALN had normal ALP serum levels. A possible explanation for this finding could be that by reducing one of the risk factors for developing NHO (high ALP levels), ALN might exert an indirect prophylactic effect. A possible downside of using ALN was the unexpected finding that patients were more prone to develop contractures while taking the drug compared to placebo [21]. Unfortunately, the study's results did not explain these adverse events' occurrence.

In contrast to earlier findings discussed above, in their meta-analysis, Yolcu et al. [23] found no statistically significant difference in NHO incidence and bisphosphonate use (p=0.58). Overall, the evidence remains inconclusive in recommending bisphosphonates as a therapeutic option for preventing NHO. Prospective studies with larger sample size and longer follow-up duration are needed to ascertain the efficacy of bisphosphonates.

NSAIDs

Local inflammation seems to play a pivotal role in the pathogenesis of NHO, and NSAIDs have been shown to prevent HO by inhibiting the differentiation of OPCs [40]. Prostaglandins are involved in regulating mesenchymal cell differentiation into osteoblasts, the bone-forming cells. One of the proposed mechanisms by which HO occurs is the excess production of prostaglandin E2 [23]. These prostaglandins may also indirectly influence the expression of bone morphogenic proteins in soft tissue, which is another factor associated with NHO [41]. NSAIDs by inhibiting cyclooxygenase (COX), lead to decreased synthesis of prostaglandins, prostacyclin, and thromboxane, thereby preventing ossification.

Studies have examined the use of indomethacin, rofecoxib, and celecoxib as prophylaxis for NHO following SCI. Banovac et al. [15,16] evaluated the efficacy of NSAIDs in preventing NHO following SCI. In their first RCT, Banovac et al. [15] randomized 33 patients post SCI and treated them prophylactically with slow-release indomethacin 75 mg daily or a placebo for three weeks. There was a significantly higher incidence of NHO in patients who received a placebo when compared with the treatment group who received indomethacin (p<0.001). Similarly, in their second RCT, Banovac et al. [16] randomized 76 patients post SCI into either the treatment group (25 mg rofecoxib daily for two weeks) or a control group. Again, a significantly lower incidence of NHO was observed in the treatment group than in the placebo group (p<0.05). 

These results reflect those of Zakrasek et al. [22], who observed that patients with SCI that had received more than 15 days of NSAID prophylaxis had an odds ratio of 0.1 of being diagnosed with NHO compared to placebo. The findings of Yolcu et al.'s [23] systematic review and meta-analysis add further credence to the effectiveness of NSAIDs in preventing NHO. While in their overall analysis, they did not find sufficient evidence to suggest a statistically significant benefit to the use of prophylactic medications in preventing NHO compared to placebo. However, when only analyzing NSAIDs, those who had received NSAIDs as prophylaxis showed a significantly lower incidence of NHO compared to placebo (p<0.001).

While NSAIDs have a relatively favorable side-effect profile, some concerns need to be addressed. For instance, rofecoxib is no longer available in the market due to its considerable cardiovascular effects. In addition, long-term NSAID use is associated with significant gastrointestinal side effects. Another concern is that NSAID use may affect fracture healing and spinal fusion, thereby limiting its use in the setting of multiple fractures [21]. However, this claim has been questioned, and NSAIDs continue to be used in spinal fusion procedures.

Limitations

This systematic review does have limitations. First, we found only a single study on NHO prophylaxis in the TBI population. Second, length of treatment and follow-up time varied significantly across all studies. Third, studies diagnosed NHO using either bone scintigraphy, plain radiographs, or clinical signs. As a result, there was a lack of a standardization tool for diagnosing NHO, thereby limiting the effective comparison of various interventions. Furthermore, most participants included in the studies were predominantly male, making it difficult to draw definite conclusions about the sex predilections of NHO. Finally, despite all nine studies being appraised as high-quality papers, only three studies were RCTs.

## Conclusions

NHO is a debilitating condition that complicates rehabilitation in patients recovering from TBI or SCI. The studies included in our review focused on the effectiveness of warfarin, PLIMF, bisphosphonates, and NSAIDs in preventing NHO. While warfarin and bisphosphonates appear to prevent NHO, the overall evidence is inconclusive. Furthermore their considerable adverse effect profile and the absence of RCTs limit their recommendation. To date, only NSAIDs and PLIMF have compelling evidence to support their use in preventing NHO based on the results of high-quality RCTs. However, undesirable side effects or feasibility constraints may hinder the clinical use of some of these interventions. Nevertheless, these adverse effects may be minor compared to the overall negative impact NHO can have on the quality of life. Given the recent advances in our understanding of NHO pathophysiology, there remains substantial potential for discovering new therapeutic approaches. We believe prospective randomized trials with larger sample sizes and longer follow-ups are needed to assess the effectiveness of alternative interventions in NHO prophylaxis.
